# Robotic Posterior Urethroplasty after Complete Urethral Transection During Abdominal Perineal Resection

**DOI:** 10.1590/S1677-5538.IBJU.2019.0425

**Published:** 2020-11-18

**Authors:** Adam S. Baumgarten, Kevin Heinsimer, Lucas Wiegand

**Affiliations:** 1 University of South Florida Department of Urology TampaFlorida United States Department of Urology, University of South Florida, Tampa General Circle, Tampa, Florida, United States;; 2 USF Health Morsani College of Medicine Department of Urology TampaFlorida United States Department of Urology, USF Health Morsani College of Medicine, Tampa General Circle, Tampa, Florida, United States

## Abstract

**Introduction and objective:**

Urethral trauma after colorectal surgery is rare, and therefore, there is a paucity of literature on their management in the current era. Additionally, there is a lack of cases describing robotic posterior urethral repair without a simultaneous perineal dissection or history of prostate cancer treatment.

**Materials and methods:**

Description of a robotic transabdominal posterior urethroplasty in a 39-year-old male with complete urethral transection after laparoscopic abdominal perineal resection (APR).

**Results:**

The patient sustained complete urethral disruption while undergoing an APR. Imaging was consistent with urologic trauma limited to a urethral transection proximal to the membranous urethra. Three days after the APR, the patient was taken to the OR for repair. Prior port sites were utilized for our robotic port placement, the retropubic space was developed and dorsal venous complex divided similarly to a prostatectomy. After identifying the urethra with the aid of a cystoscope, the prostatic urethra was anastomosed to the membranous using a 3-0 barbed monofilament. At postoperative week four, a voiding cystourethrogram showed a small leak, therefore the urethral catheter was left for a total of six weeks. At last follow-up, the patient was voiding per urethra without fistula, incontinence, or stricture.

**Conclusions:**

Early robotic repair of an iatrogenic posterior urethral disruption is feasible with successful short-term outcomes. This is a select and rare complication of colorectal surgery and therefore, long-term stricture free rates are yet to be determined.

## ARTICLE INFO

**Available at:**http://www.intbrazjurol.com.br/video-section/20190425_Baumgarten_et_al

